# Structure of the Prx6-subfamily 1-Cys peroxiredoxin from *Sulfolobus islandicus*

**DOI:** 10.1107/S2053230X19006472

**Published:** 2019-05-13

**Authors:** Sander Stroobants, Inge Van Molle, Queen Saidi, Karl Jonckheere, Dominique Maes, Eveline Peeters

**Affiliations:** a Structural Biology Brussels, Department of Bioengineering Sciences, Vrije Universiteit Brussel, Pleinlaan 2, B-1050 Brussels, Belgium; b VIB–VUB Center for Structural Biology, Pleinlaan 2, B-1050 Brussels, Belgium; c Research Group of Microbiology, Department of Bioengineering Sciences, Vrije Universiteit Brussel, Pleinlaan 2, B-1050 Brussels, Belgium

**Keywords:** peroxiredoxins, archaea, *Sulfolobus islandicus*, Prx6, chaperones

## Abstract

Aerobic thermoacidophilic archaea belonging to the genus *Sulfolobus* harbor peroxiredoxins, thiol-dependent peroxidases that assist in protecting the cells from oxidative damage. Here, the crystal structure of the 1-Cys peroxiredoxin from *Sulfolobus islandicus*, named 1-Cys SiPrx, is presented. A 2.75 Å resolution data set was collected from a crystal belonging to space group *P*2_1_2_1_2_1_, with unit-cell parameters *a* = 86.8, *b* = 159.1, *c* = 189.3 Å, α = β = γ = 90°. The structure was solved by molecular replacement using the homologous *Aeropyrum pernix* peroxiredoxin (ApPrx) structure as a search model. In the crystal structure, 1-Cys SiPrx assembles into a ring-shaped decamer composed of five homodimers. This quaternary structure corresponds to the oligomeric state of the protein in solution, as observed by size-exclusion chromatography. 1-Cys SiPrx harbors only a single cysteine, which is the peroxidatic cysteine, and lacks both of the cysteines that are highly conserved in the C-terminal arm domain in other archaeal Prx6-subfamily proteins such as ApPrx and that are involved in the association of dimers into higher-molecular-weight decamers and dodecamers. It is thus concluded that the *Sulfolobus* Prx6-subfamily protein undergoes decamerization independently of arm-domain cysteines.

## Abstract 2

The crystal structure of the 1-Cys peroxiredoxin from *S. islandicus* is presented; it assembles into a ring-shaped decamer composed of five homodimers. It is concluded that this Prx6-like protein undergoes decamerization independently of arm-domain cysteines.

## 1. Introduction   

All organisms need to cope with the presence of reactive oxygen species (ROS) that threaten cellular integrity by damaging nucleic acids, proteins, lipids and other biomolecules. This is certainly the case for thermoacidophilic archaea belonging to the Sulfolobales that live in volcanic hot springs (Brock *et al.*, 1972 ▸), which are strongly oxidative habitats. Furthermore, these organisms have a metabolism that is obligately dependent on aerobic respiration and thus generates an additional excessive amount of toxic ROS as by­products (She *et al.*, 2001 ▸; Chen *et al.*, 2005 ▸). Therefore, *Sulfolobus* species harbor a variety of defensive enzymes and antioxidant molecules that are responsible for the detoxification of ROS. Despite the absence of catalase enzymes, antioxidant enzymes named peroxiredoxins (Prxs), which are ubiquitously present in all three domains of life (Schröder & Ponting, 1998 ▸; Wood *et al.*, 2003 ▸), constitute an important element of the ROS defense system in *Sulfolobus* spp. (Limauro *et al.*, 2006 ▸). Prx proteins reduce hydrogen peroxide and organic peroxides to water and the corresponding alcohols, respectively, by making use of a thiol-dependent mechanism involving catalytic cysteine residues (Wood *et al.*, 2003 ▸). The diverse family of Prxs has been divided into six subfamilies based on active-site profiling (Soito *et al.*, 2011 ▸). Archaeal Prxs belong to one of two subfamilies, BCP/PrxQ or Prx6 (Soito *et al.*, 2011 ▸), with the latter subfamily typically being 1-Cys Prxs that are characterized by a peroxidatic cysteine residue (C_P_), which is the sole redox-active cysteine, and thus lacking the resolving cysteine (C_R_) that is present in 2-Cys Prxs and forms a disulfide bond to the oxidized C_P_.

**Figure 1:**
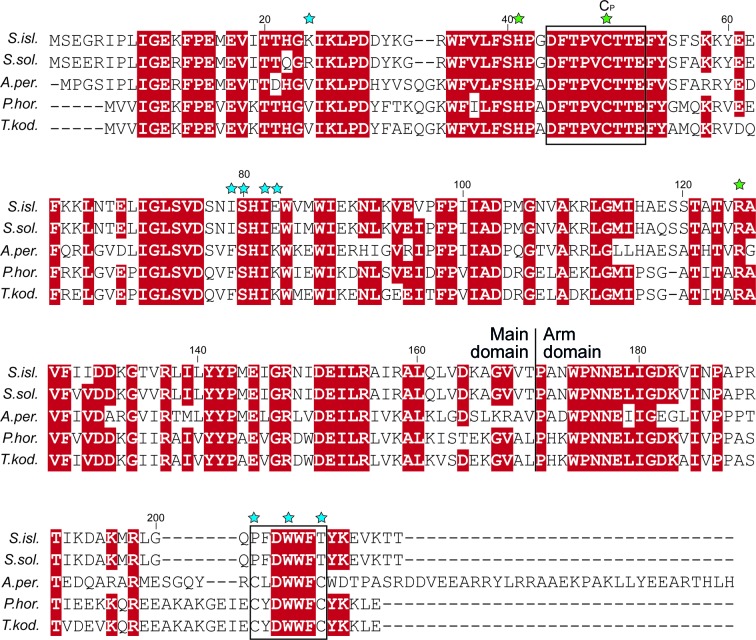
Amino-acid sequence alignment of archaeal Prx6-subfamily 1-Cys peroxiredoxins. *S.isl*, *Sulfolobus islandicus* REY15a (ADX84439.1); *S.sol.*, *Sulfolobus solfataricus* P2 (P95895; 95% sequence identity); *A.per.*, *Aeropyrum pernix* K1 (Q9Y9L0.1; 61% sequence identity); *P.hor.*, *Pyrococcus horikoshii* OT3 (NP_143112.1; 57% sequence identity); *T.kod.*, *Thermococcus kodakarensis* KOD1 (YP_182950.1; 55% sequence identity). (Highly) conserved residues are indicated in red and conserved motifs are boxed. Residues that are important for peroxidase activity are indicated with a green star (the peroxidatic cysteine is labeled C_P_), while residues shown to be involved in (redox-sensitive) oligomerization of the protein are indicated with a blue star. Sequence alignment was performed with *ClustalW* (Larkin *et al.*, 2007 ▸) and graphically prepared with *Adobe Illustrator*.

*Sulfolobus* species generally harbor four different Prxs, originally named Bcp1, Bcp2, Bcp3 and Bcp4 (Limauro *et al.*, 2006 ▸), with Bcp2 belonging to the Prx6 subfamily of 1-Cys Prxs and the others being BCP/PrxQ members (Table 1 ▸; Soito *et al.*, 2011 ▸). All four Prxs have been experimentally characterized in *S. solfataricus* (Limauro *et al.*, 2006 ▸, 2008 ▸, 2010 ▸). The 1-Cys Prx Prx6 member is highly conserved in archaea, with 61%, 57% and 55% sequence identity between *S. islandicus* Bcp2 (here named 1-Cys SiPrx) and its orthologs from *Aeropyrum pernix* (ApPrx), *Pyrococcus horikoshii* (PhPrx) and *Thermococcus kodakarensis* (TkPrx), respectively (Fig. 1 ▸). Furthermore, there is a significant degree of identity to 1-Cys Prxs from bacteria, humans, *Arabidopsis* and yeast cells (Lee *et al.*, 2015 ▸). In contrast to archaeal 2-Cys Prxs from the BCP/PrxQ subfamily, such as Bcp1 and Bcp4 from *Sulfolobus* spp., 1-Cys Prxs such as TkPrx and *S. solfataricus* Bcp2 are not constitutively expressed but instead display an induced expression level in response to oxidative stress (Limauro *et al.*, 2006 ▸; Lee *et al.*, 2015 ▸). The conserved N-terminal cysteine has been shown to be the peroxidatic C_P_ responsible for H_2_O_2_ detoxification (Limauro *et al.*, 2006 ▸; Lee *et al.*, 2015 ▸; Fig. 1 ▸). Besides peroxidase activity, archaeal 1-Cys Prxs are moonlighting enzymes that also have a chaperone activity, thereby protecting proteins and DNA from damage caused by oxidative and thermal stress (Limauro *et al.*, 2006 ▸; Lee *et al.*, 2015 ▸). For TkPrx, it has been shown that two C-terminal cysteines are responsible for a redox-dependent switch between a dimeric and a higher oligomeric state, with the former mainly functioning as a peroxidase and the latter mainly performing a molecular chaperone function (Lee *et al.*, 2015 ▸). Intriguingly, while these C-terminal cysteines are highly conserved in all other archaeal 1-Cys Prxs, the *Sulfolobus* Prx6-subfamily 1-Cys Prx proteins lack both of them and only have a single cysteine that is the C_P_ (Fig. 1 ▸), thus evoking questions about its oligomeric state.

ApPrx and PhPrx have been structurally characterized (Mizohata *et al.*, 2005 ▸; Nakamura *et al.*, 2006 ▸, 2010 ▸, 2013 ▸). In contrast, no structural information was available about this Prx enzyme in *Sulfolobus* spp. Here, we describe the crystallization and structure determination of 1-Cys SiPrx from *S. islandicus*, a highly conserved homolog (95% sequence identity) of Bcp2 from the closely related *S. solfataricus*.

## 2. Materials and methods   

### 2.1. Heterologous expression and purification of 1-Cys SiPrx   

The coding sequence for *S. islandicus* 1-Cys SiPrx (*SiRe_0346*) was cloned into the NcoI and XhoI restriction sites of pET-28b to generate the pET-28bxSiRe_0346 expression vector (Table 2 ▸). *Escherichia coli* Rosetta cells were transformed with this plasmid vector and cultivated in lysogeny broth (LB) medium supplemented with 60 µg ml^−1^ kanamycin at 310 K. The recombinant expression of the C-terminally His-tagged protein was induced by the addition of 0.1 m*M* isopropyl β-d-1-thiogalactopyranoside (IPTG) at an optical density (OD_600 nm_) of 0.7, followed by further incubation at 310 K overnight. After centrifugation, the cells were resuspended in buffer *A* (20 m*M* Na_2_HPO_4_, 500 m*M* NaCl, 40 m*M* imidazole pH 7.4) and lysed by sonication for 15 min at 20% of the maximal amplitude (750 W VibraCell sonicator, Bioblock Sciences). After centrifugation for 10 min at 15 000*g*, the supernatant was loaded onto a 5 ml HisTrap FF column (GE Healthcare) equilibrated with buffer *A* and the protein was eluted using a linear gradient to 500 m*M* imidazole in the same buffer using an ÄKTA FPLC system (GE Healthcare). Finally, dialysis was performed against 10 m*M* Tris–HCl pH 7.5 or 50 m*M* Na_2_HPO_4_ pH 7.4, 150 m*M* NaCl, depending on the downstream analysis.

### 2.2. Crystallization   

Screening for crystallization conditions was performed by sitting-drop vapor diffusion in two 96-well plates using the Index HT (Hampton Research) and JCSG-*plus* (Molecular Dimensions) screens at 293 K. Various conditions led to crystallization, with most containing MgCl_2_·6H_2_O as the precipitant and PEG as a crowding agent. Optimization of the crystallization conditions of 1-Cys SiPrx was performed by the hanging-drop vapor-diffusion method, in which 2 µl protein solution (7.5 mg ml^−1^ in 10 m*M* Tris–HCl pH 7.5) was mixed with 2 µl reservoir solution and equilibrated against 200 µl of the same reservoir solution, followed by incubation at 283 K. The optimal reservoir solution was found to be 10 m*M* Tris–HCl pH 7.5, 15%(*w*/*v*) PEG 1500, 0.1 *M* MgCl_2_. Using this condition, diffracting crystals were obtained after a two-week incubation.

### 2.3. Data collection, processing and structure determination   

1-Cys SiPrx crystals were submerged in the original reservoir solution with 35%(*w*/*v*) PEG 1500 for cryoprotection and flash-cooled in liquid nitrogen at 100 K. Diffraction data were collected on beamline ID29 at the ESRF, Grenoble, France using a PILATUS 6M detector. Data sets were obtained from four different crystals, but only data from a single crystal were used for structure determination. The first 1500 images of the collected data were processed using *AutoPROC* (Vonrhein *et al.*, 2011 ▸). As the diffraction data were found to be anisotropic, *STARANISO* (Tickle *et al.*, 2018 ▸) was used to process and scale the final data. The elliptical resolution cutoffs for the a*, b* and c* axes were determined to be 3.65, 2.76 and 2.55 Å, respectively. Based on this information, a resolution cutoff of 2.75 Å was selected. Consequently, the spherical completeness is low both overall and in the highest resolution shell. However, the completeness exceeds 95% in all shells up to a resolution of 3.4 Å.

Molecular replacement was performed with *Phaser* (McCoy *et al.*, 2007 ▸), as included in the *PHENIX* suite (Adams *et al.*, 2010 ▸), using the ApPrx structure from *A. pernix* (PDB entry 3a2v; Nakamura *et al.*, 2010 ▸) with 61% sequence identity as a model. An ApPrx monomer was used as the search model and the first round of the search resulted in an asymmetric unit with ten monomer copies. The initial SiPrx model was created using *AutoBuild* (Adams *et al.*, 2010 ▸) and was further built manually using *Coot* (Emsley *et al.*, 2010 ▸). Refinement was performed with *PHENIX* (Adams *et al.*, 2010 ▸) using non­crystallographic constraints and TLS refinement. The quality of the structure was analyzed with *MolProbity* (Chen *et al.*, 2010 ▸). Given the lack of electron density at the N-terminal and C-terminal ends, the first four and the last two residues were not modeled, including the His tag. The data-collection and refinement statistics are summarized in Table 3 ▸. The crystal structure has been deposited in the Protein Data Bank (PDB) with accession code 6q5v. All figures displaying protein structures were prepared with *PyMOL* (DeLano, 2002 ▸) and *UCSF Chimera* 1.12 (Pettersen *et al.*, 2004 ▸).

### 2.4. Size-exclusion chromatography   

For size-exclusion chromatography (SEC), about 2 mg of 1-Cys Prx protein was mixed with 0.5 mg blue dextran and migrated on a Superdex 200 column (GE Healthcare) using an ÄKTA FPLC system (GE Healthcare) with 50 m*M* Na_2_HPO_4_ pH 7.4, 150 m*M* NaCl as the running buffer. The following proteins were used to obtain the molecular-weight standard curve: thyroglobulin (670 kDa), γ-globulin (158 kDa), ovalbumin (44 kDa) and myoglobulin (17 kDa).

## 3. Results and discussion   

### 3.1. Structure of 1-Cys SiPrx   

**Figure 2:**
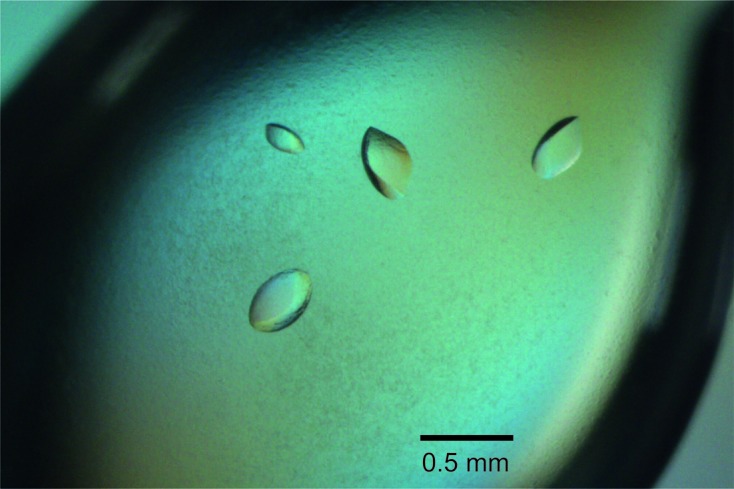
Crystals of *S. islandicus* 1-Cys SiPrx.

**Figure 3:**
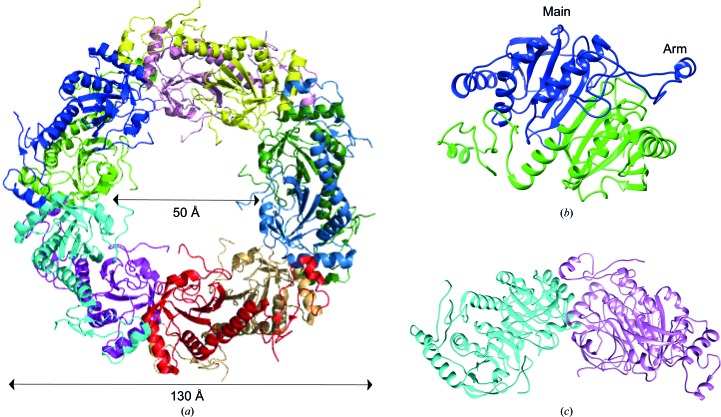
Structure of 1-Cys SiPrx. (*a*) Decameric structure, with each monomer colored differently and with the average dimensions indicated. (*b*) Dimer structure, with each monomer colored differently and with the main and arm-domain regions indicated. (*c*) Dimer–dimer interface, with each dimer colored differently to emphasize the dimer–dimer interactions.

Crystals of 1-Cys SiPrx appeared after 30 days of incubation using the hanging-drop vapor-diffusion method (Fig. 2 ▸). The crystal structure of 1-Cys SiPrx was determined at 2.75 Å resolution, with *R*_cryst_ and *R*_free_ values of 21.3 and 22.8%, respectively (Table 3 ▸; Fig. 3 ▸). The asymmetric unit contained ten subunits arranged in a symmetrical toroid-shaped ring of five dimers (Fig. 3 ▸ *a*). Two domains can be discerned in the monomeric structure: an N-terminal main domain and a smaller C-terminal arm domain (Fig. 3 ▸ *b*). The protein dimerizes with an extensive monomer–monomer interface created between the main domains involving the formation of a central antiparallel β-sheet, whereas the two arm domains stick out of the dimeric structure (Fig. 3 ▸ *b*). In this structure, 21% of the total accessible surface area of a monomer is buried upon dimerization. Dimer–dimer interactions consist of contacts both between main domains and between adjacent main and arm domains (Fig. 3 ▸ *c*).

**Figure 4:**
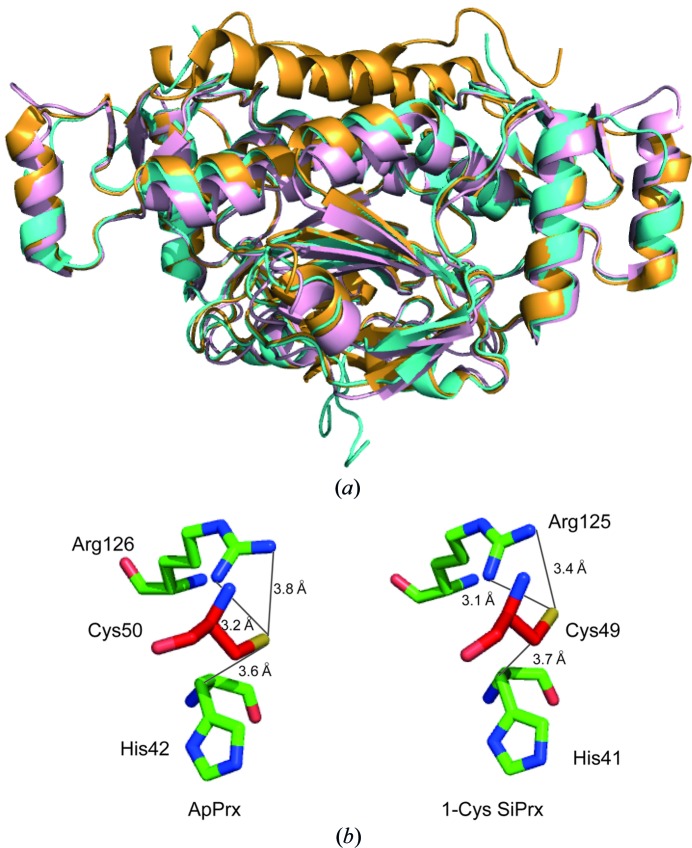
Structural alignment of 1-Cys SiPrx with other archaeal Prxs. (*a*) Superimposition of 1-Cys SiPrx (blue; PDB entry 6q5v), ApPrx (orange; PDB entry 3a2v) and PhPrx (pink; PDB entry 3w6g) with the N-terminal ends oriented downwards and the C-terminal ends oriented upwards. (*b*) Enlarged image displaying the conformations of the active-site residues in ApPrx and 1-Cys SiPrx. The peroxidatic C_P_, which is oriented in front of the other two residues, is colored red and the distances to its S atom are indicated.

The quaternary structure of 1-Cys SiPrx is similar to those of PhPrx and ApPrx (Mizohata *et al.*, 2005 ▸; Nakamura *et al.*, 2006 ▸, 2013 ▸; Lee *et al.*, 2015 ▸). The dimensions of the decameric ring agree well with those observed for these homologous archaeal Prxs, which also form decameric rings (Fig. 3 ▸ *a*). A superimposition demonstrates the similarity of the monomeric 1-Cys SiPrx, PhPrx and ApPrx structures, with the exception of the C-terminal tail that is unique to ApPrx and is absent in 1-Cys SiPrx (Fig. 4 ▸ *a*). The active site, formed by the peroxidatic Cys49, His41 and Arg125, has the same conformation as those of ApPrx and PhPrx (Fig. 4 ▸ *b*). In contrast to the ApPrx structure, in which citrate was present in the active site (Nakamura *et al.*, 2013 ▸), the H_2_O_2_-binding pocket of 1-Cys SiPrx was found to be empty.

### 3.2. Oligomeric state of 1-Cys SiPrx in solution   

**Figure 5:**
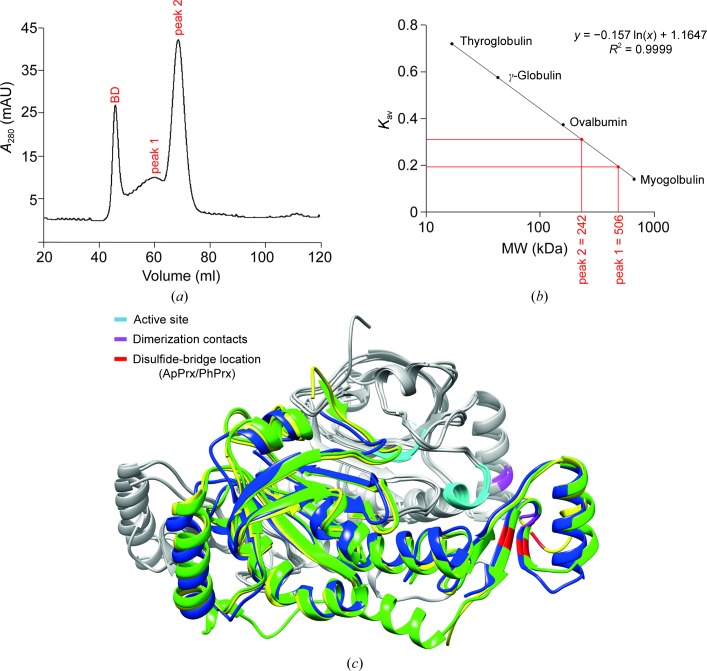
Oligomeric state of 1-Cys SiPrx in solution and the residues involved in oligomerization. (*a*) SEC chromatogram indicating the peak corresponding to blue dextran (BD), which was used for determination of the void volume *V*_0_, and 1-Cys SiPrx peaks 1 and 2. (*b*) Calibration curve used for the calculation of the molecular weights of the 1-Cys SiPrx peaks. (*c*) Superimposition of dimeric portions of the 1-Cys SiPrx, ApPrx and PhPrx structures. For each dimer, one monomer is depicted in gray and the other in yellow (SiPrx), blue (ApPrx) or green (PhPrx). Residues that are involved in crucial functions of the protein are colored as indicated.

SEC analysis demonstrated that in solution 1-Cys SiPrx exists as a heterogeneous population of a major 242 kDa species (peak 1) and a minor 506 kDa species (peak 2) (Figs. 5 ▸ *a* and 5 ▸ *b*). Taking into account the monomeric molecular weight of 24.7 kDa, we conclude that 1-Cys SiPrx mainly exists in a decameric oligomeric form corresponding to the quaternary structure observed in the crystal structure, and that a smaller population exists of a higher-order oligomer, which is probably a 20-subunit protein. ApPrx, PhPrx and TkPrx also form decameric oligomeric states in solution in untreated (non­reducing) conditions (Nakamura *et al.*, 2006 ▸, 2013 ▸; Lee *et al.*, 2015 ▸). TkPrx forms dimers in reducing conditions, while for both TkPrx and PhPrx it has been shown that overoxidation leads to dodecameric structures (Lee *et al.*, 2015 ▸; Nakamura *et al.*, 2017 ▸).

Archaeal 1-Cys Prxs are characterized by a C*X*DWWFC motif in the C-terminal arm domain (Mizohata *et al.*, 2005 ▸; Lee *et al.*, 2015 ▸; Nakamura *et al.*, 2017 ▸). Both cysteines are highly conserved in the archaeal enzymes and contribute to the redox-dependent association of dimers into higher-order oligomers (Lee *et al.*, 2015 ▸; Nakamura *et al.*, 2017 ▸). This is exemplified by mutational analysis of TkPrx: upon mutating one of the two cysteines to a serine, the oligomeric state of the protein becomes dimeric instead of decameric (Lee *et al.*, 2015 ▸). Furthermore, upon overoxidation by H_2_O_2_ treatment, leading to the dodecameric form, an intramolecular disulfide bridge is established between these cysteines in PhPrx (Nakamura *et al.*, 2017 ▸). It could be hypothesized that disulfide-bridge formation converts oxidative stress signals into subtle conformational changes that affect the oligomeric state. Exceptionally, *Sulfolobus* 1-Cys Prx lacks both cysteines but nevertheless forms a decamer, suggesting that it has lost the redox-sensing function and that it is capable of forming higher oligomeric states independently of the arm-domain cysteines.

Besides the two cysteine residues, all other residues that contribute to transitions between dimers and higher oligomeric states (Nakamura *et al.*, 2017 ▸) are conserved or have similar properties, with the exception of the residue at the position corresponding to Lys24: ApPrx and PhPrx harbor a valine at the homologous position.

In conclusion, although untreated 1-Cys SiPrx behaves mainly as a decamer, like other archaeal Prx6-subfamily proteins, it could be postulated that the protein is less sensitive to redox signaling for oligomeric state conversions and that the decamer is the dominant form. Since it has been shown for TkPrx that higher nondimeric oligomeric forms function not only as peroxidases but mostly as chaperones (Lee *et al.*, 2015 ▸), it could be hypothesized that the chaperone function of 1-Cys SiPrx is more specialized compared with that of TkPrx.

## Supplementary Material

PDB reference: 1-Cys peroxiredoxin, 6q5v

## Figures and Tables

**Table 1:** Overview of the genes encoding peroxiredoxins in *Sulfolobus* spp

Name in *S. solfataricus* (Limauro *et al.*, 2006 ▸)	Prx subfamily (Soito *et al.*, 2011 ▸)	*S. islandicus* REY15a	*S. solfataricus* P2	*S. acidocaldarius* DSM639	*S. tokodaii* strain 7
Bcp1	BCP/PrxQ	*SiRe_0725*	SSO2071	*Saci_2227*	ST0721
Bcp2	Prx6	*SiRe_0346*	SSO2121	—	ST2442
Bcp3	BCP/PrxQ	*SiRe_0067*	SSO2255	*Saci_0058*	ST2102
Bcp4	BCP/PrxQ	*SiRe_2592*	SSO2613	*Saci_1125*	ST1785

**Table 2:** Macromolecule production

Source organism	*S. islandicus* REY15a
DNA source	Genomic DNA
Forward primer	5′-CATGCCATGGTGAGTGAGGGAAGAATTCCATTAATAG-3′
Reverse primer	5′-CCGCTCGAGTGTCGTTTTAACTTCTTTATAAGTGAAC-3′
Cloning vector	pET-28b (Novagen)
Expression vector	pET-28b (Novagen)
Expression host	*E. coli* Rosetta
Complete amino-acid sequence of the construct produced	MVSEGRIPLIGEKFPEMEVITTHGKIKLPDDYKGRWFVLFSHPGDFTPVCTTEFYSFSKKYEEFKKLNTELIGLSVDSNISHIEWVMWIEKNLKVEVPFPIIADPMGNVAKRLGMIHAESSTATVRAVFIIDDKGTVRLILYYPMEIGRNIDEILRAIRALQLVDKAGVVTPANWPNNELIGDKVINPAPRTIKDAKMRLGQPFDWWFTYKEVKTTLEHHHHHH

**Table 3:** Data-collection and refinement statistics Values in parentheses are for the outer shell.

Data collection	
X-ray source	ID29, ESRF
Detector	PILATUS 6M
Wavelength (Å)	0.976
Space group	*P*2_1_2_1_2_1_
Unit-cell parameters (Å, °)	*a* = 86.8, *b* = 159.1, *c* = 189.3, α = 90.0, β = 90.0, γ = 90.0
Resolution range, spherical (Å)	76.19–2.75 (2.86–2.75)
Resolution range, ellipsoidal (Å)	*a* = 3.65, *b* = 2.76, *c* = 2.55
*R*_merge_	0.095 (0.957)
Completeness, spherical (%)	74.6 (33.0)
Completeness, ellipsoidal (%)	94.5 (93.9)
Total reflections	285938 (14834)
Unique reflections	51577 (2580)
Multiplicity	5.5 (5.7)
〈*I*/σ(*I*)〉	12.7 (1.9)
Wilson *B* factor (Å^2^)	66.2
CC_1/2_	0.999 (0.776)
Refinement	
Resolution range (Å)	76.2–2.75
No. of reflections	51522 (2221)
*R*_cryst_/*R*_free_ (%)	21.3 (38.6)/22.8 (40.6)
Protein atoms	16820
Water molecules	0
R.m.s.d., bond lengths (Å)	0.102
R.m.s.d., bond angles (°)	1.60
Average *B* factor (Å^2^)	62.0
Ramachandran plot, residues in (%)	
Favored region	93.2
Allowed region	6.8
